# Effect of m^6^A Recognition Protein YTHDC1 on Skeletal Muscle Growth

**DOI:** 10.3390/ani15131978

**Published:** 2025-07-05

**Authors:** Huijun Huang, Geyan Lu, Liyao Xiao, Baohua Tan, Yuming Yang, Linjun Hong, Zicong Li, Gengyuan Cai, Ting Gu

**Affiliations:** 1 National Engineering Research Center for Breeding Swine Industry, College of Animal Science, South China Agricultural University, Guangzhou 510642, China; 1227704881@stu.scau.edu.cn (H.H.); lgy@stu.scau.edu.cn (G.L.); xlyao@stu.scau.edu.cn (L.X.); tanbaohua@stu.scau.edu.cn (B.T.); yym012209@stu.scau.edu.cn (Y.Y.); linjun.hong@scau.edu.cn (L.H.); lizicong@scau.edu.cn (Z.L.); cgy0415@scau.edu.cn (G.C.); 2 State Key Laboratory of Swine and Poultry Breeding Industry, College of Animal Science, South China Agricultural University, Guangzhou 510642, China; 3 Gene Bank of Guangdong Local Livestock and Poultry, College of Animal Science, South China Agricultural University, Guangzhou 510642, China

**Keywords:** m^6^A modification, YTHDC1, skeletal muscle growth, alternative splicing

## Abstract

Skeletal muscle is the largest heterogeneous organ in the body, and multiple factors in intrinsic genetic and epigenetic regulation influence its growth. The N^6^-methyladenosine ed(m^6^A) modification is a conserved and most prevalent RNA modification, whose function is dependent on m^6^A writers, erasers, and m^6^A readers, such as the YTH protein family. YTHDC1 is the only member of the YTH protein family member that exists in the cell nucleus, which plays an important role in mRNA alternative polyadenylation and alternative splicing processes. However, the function of YTHDC1 in regulating myoblast proliferation, differentiation, and in vivo skeletal muscle development remains unclear. Therefore, in this study, we studied the function of YTHDC1 in C2C12 cell line and mouse. Our results showed that YTHDC1 significantly promoted myogenic differentiation while inhibiting myoblast proliferation in C2C12 cells, and the results of our in vivo experiment showed that interfering with YTHDC1 led to a significant enhancement of muscle growth in mice. Furthermore, the transcriptome sequencing analysis revealed that YTHDC1 might modulate skeletal muscle development by regulating alternative splicing of genes, including *Akap13*, *Smarca2*, *Tnnt3*, and *Neb*. Our study shed light on understanding the function and molecular mechanisms of YTHDC1 in regulating skeletal muscle development, highlighting the critical contribution of m^6^A-mediated RNA splicing in muscle growth. These results indicated that YTHDC1 could be a potential breeding target gene to enhance meat quality in livestock.

## Simple Summary

YTHDC1 is the only member of the YTH protein family that exists in the cell nucleus, which plays an important role in the Messenger RNA (mRNA) alternative splicing process. Therefore, this study aimed to systematically analyze the impact of m6A recognition protein YTHDC1 in myoblast proliferation, differentiation, and skeletal muscle growth in mice through both cellular and in vivo experiments combined with transcriptome sequencing.

## 1. Introduction

Muscle tissues in livestock and poultry are classified into smooth, skeletal, and cardiac muscles, and are the largest tissues in the body, constituting 40–60% of body weight. The skeletal muscle is an important organ not only for motion, but also for regulating glucose metabolism and maintaining blood glucose homeostasis, while it is the primary source of meat products in livestock and poultry as well [1]. Various intrinsic factors, including genetic and epigenetic mechanisms orchestrate and regulate skeletal muscle growth. Early research has primarily focused on the effects of individual regulatory factors or genes on skeletal muscle growth, including muscle differentiation antigens (MyoD and MyoG), myogenic regulatory factors (Myf6), muscle regulatory factors, and myocyte enhancer factor 2 [2]. However, approximately 98% of all transcripts in eukaryotes consist of non-coding RNAs, with only a small fraction of genes encoding proteins that directly participate in regulatory processes [3]. With advances in research methods, skeletal muscle growth and development regulating factors have expanded from a single-gene level to more complex signaling pathways and epigenetic regulatory mechanisms. These mechanisms include DNA methylation, histone modifications, and miRNAs, among which N^6^-methyladenosine (m^6^A) is the most prevalent and abundant RNA methylation modification.

m^6^A is a methyl modification added to the nitrogen atom at the sixth position of adenine by the m^6^A methylase complex (METTL3, METTL14, and WTAP) using S-adenosylmethionine as the methyl donor. This modification is primarily located in the 3′ untranslated region (3′ UTR), long exons, around stop codons, and near the transcription start site of the mRNA [4,5]. The m^6^A methylation modification is the most extensively studied mRNA modification [6,7]. Its function depends on the co-operation of m^6^A methylases (writers), demethylases (erasers), and m^6^A readers, which preferentially recognize and bind to m^6^A sites on the mRNA [8]. m^6^A readers include the YTH, IGF2BP, and heterogeneous nuclear ribonucleoprotein (hnRNP) families [9]. Among them, YTH protein family members YTHDF1, YTHDF3, and YTHDC1 can enhance the translation efficiency of m^6^A-modified transcripts by recruiting translation initiation factors [10,11]. YTHDC1, the only protein in the nucleus with the ability to influence RNA post-transcriptional modifications, can bind to translation initiation factors, including eIF factors or dephosphorylated SRSF3 (serine/arginine-rich splicing factor 3), to promote the initiation of translation of m^6^A-modified genes [12,13]. It also enriches m^6^A-modified mRNA on ribosomes, enhancing translation efficiency and promoting mRNA export from the nucleus for translation or degradation [13,14,15,16,17]. YTHDC1 is involved in transcriptional regulation and closely associated with splicing factors, including SR proteins, and the 3′ end processing of precursor RNAs [12]. Studies have reported that YTHDC1 deletion in mouse embryos leads to defects in alternative polyadenylation and splicing processes in oocytes, causing abnormalities in the embryo [18,19,20,21]. Extensive research has been conducted on the regulatory mechanisms of m^6^A modification in many physiological diseases, revealing that m^6^A modification regulates disease development and is involved in skeletal muscle cell proliferation and myotube formation as well [22]. Studies have demonstrated that distinct m^6^A modification profiles can be observed during the proliferation and differentiation of C2C12 cells. The overall m^6^A levels decrease during the transition from proliferation to differentiation, regulated by METTL3, which modulates m^6^A levels in myocytes to control the transition of cells [23,24,25]. Numerous muscle-specific m^6^A modification sites have been identified in mammals. For instance, in the longissimus dorsi muscle of Landrace pigs (lean-type breed), the expression levels of m^6^A at protein and mRNA levels are significantly higher than those in Jinhua pigs (obese-type breed) [26].

Alternative splicing is a post-transcriptional process in which mRNA is spliced into different fragments, generating various mRNA isoforms, and then transcribed to different protein isoforms [27]. Studies have indicated that selective splicing generates various proteins corresponding to several splice forms, influencing protein localization, stability, enzyme activity, and other properties by altering the amino acid composition of the protein. Furthermore, alternative splicing events occurring in the UTRs can directly affect transcript stability or translation efficiency, thereby regulating protein expression levels by modulating transcript abundance [28]. Alternative splicing contributes to transcriptome variation in embryonic stem cells (ESCs), which may play a regulatory role in lineage-specific differentiation processes [29,30]. Hepatotoxic regeneration-inducing agents downregulate the epithelial splicing regulatory protein 2 (ESRP2), thereby promoting the expression of “neonatal” splicing isoforms that subsequently inhibit the Hippo signaling pathway, which affects cell proliferation, differentiation, and tissue homeostasis [31]. More and more studies have demonstrated that alternative splicing plays an important role in cell differentiation, tissue development, and organ function.

Current studies indicate that m^6^A can regulate muscle development [32], but the detailed mechanism of its recognition protein YTHDC1 in regulating skeletal muscle growth remains unknown. Therefore, this study aimed to systematically analyze the effect of m^6^A recognition protein YTHDC1 in myoblast proliferation, differentiation, and skeletal muscle growth in mice through both cellular and in vivo experiments combined with transcriptome sequencing. This study will enhance the comprehension of the molecular mechanisms governing skeletal muscle development and further elucidate and refine the regulatory mechanisms of skeletal muscle growth. It also offers novel insights and directions for the genetic improvement and breeding of meat-type animals.

## 2. Materials and Methods

### 2.1. Experimental Cells and Animals

The mouse C2C12 myoblast cell line cells used in this study were acquired from the Cell Resource Center (Shanghai Institutes for Biological Sciences, Shanghai, China). Twelve six-week-old male C57BL/6 (C57) mice were obtained from Jinwei (Guangzhou, China). The mice were maintained under hygienic conditions at 20–26 °C, with a relative humidity of 50–60%, and were provided with feed weekly. All animal experiments in this study were approved by the Ethics Committee of the Experimental Animal Center, South China Agricultural University (Permit Number 2021F036, Permit Date 2 March 2021).

### 2.2. Synthesis of siRNA Interfering Fragments and Overexpression Vectors

siRNA-interfering fragments targeting mouse YTHDC1 and a pair of control siRNAs (siRNA-NC) were designed and synthesized by GenePharma (Shanghai, China). (sequences are listed in the Table S1). The overexpression vector was constructed by downloading the CDs sequence of YTHDC1 (accession number: CCDS84898.1) from the NCBI (National Center for Biotechnology Information) website, which was then constructed into a pcDNA3.1(+) backbone vector (Figure S1) by Tsingke (Guangzhou, China).

### 2.3. Cell Transfection

Mouse C2C12 myoblast cells were cultured in DMEM (Dulbecco’s Modified Eagle Medium) containing 10% fetal bovine serum (Gibco, Grand Island, NY, USA) at 37 °C in 5% CO_2_. When C2C12 cells reached 70–80% confluence, transfection was performed in a six-well plate. For plasmid transfection using Lipofectamine 3000 (L3000075, Thermo Fisher Scientific, Waltham, MA, USA), the prepared solutions A and B (Table 1) were gently mixed thoroughly. Among them, Opti-MEM is Opti-MEM serum reducing medium, P3000 is Lipofectamine^TM^ P3000. The mixture was then incubated at room temperature for 15 min. The medium in each well was replaced with 2 mL of fresh growth medium, and 250 μL of the transfection mixture was added to each well. The plate was gently swirled to ensure an even distribution of the mixture. After 6 h of incubation, the medium was replaced with a fresh growth medium. For transfection of interfering fragments using jetPRIME (101000046, Polyplus-transfection S.A, Illkirch-Graffenstaden, France), the transfection mixture was prepared by combining 17.5 μL siRNA/NC, 700 μL jetPRIME Buffer and 1 μL jetPRIME reagent, and the mixture was allowed to stand at room temperature for 10–15 min. The subsequent steps were the same as those for Lipofectamine 3000.

### 2.4. Real-Time Fluorescent Quantitative PCR Detection of Gene mRNA Expression

Total RNA was extracted from the interfered or overexpressed C2C12 cells using a TRIzol reagent (15596026CN, Invitrogen, Carlsbad, CA, USA). Total RNA was reverse transcribed into cDNA using a reverse transcription kit (Vazyme Biotech, Nanjing, China). Real-time fluorescent quantitative PCR was used to detect the mRNA expression levels of the proliferation marker gene Pcna and the muscle proliferation marker gene Ki67 in proliferating C2C12 cells. For cells differentiated for three days, real-time fluorescent quantitative PCR was used to measure the mRNA expression levels of differentiation marker genes MyoG, MyhC, and MyoD (sequences are listed in Table S1).

### 2.5. Western Blotting Detection of Protein Expression

Total protein from C2C12 cells was extracted using RIPA (Radio Immunoprecipitation Assay) cell lysis buffer containing 1% Phenylmethanesulfonyl fluoride (ST506, Beyotime, Shanghai, China). After adding 6× protein loading buffer (Beyotime Biotechnology, Shanghai, China), the samples were incubated at 95 °C for 10 min. Approximately 20 μL of each sample was loaded onto a 10% polyacrylamide gel for electrophoresis (constant voltage of 150 V). After electrophoresis, the proteins were transferred to a Polyvinylidene Fluoride (PVDF) membrane using the wet transfer method at 200 mA. The PVDF membrane was washed twice with Tris-Buffered Saline (TBS) and blocked with 5% non-fat milk for at least 2.5 h. After washing with TBS, the membrane was incubated overnight at 4 °C with primary antibodies: MyoG (1:500), MyoD (1:500), MyhC (1:500), Ki67 (1:2000), and Pcna (1:3000). The membrane was then incubated with HRP (Horseradish Peroxidase) -conjugated goat anti-mouse IgG (1:5000) as the secondary antibody at 37 °C for 1 h, followed by washing with Tris-Buffered Saline with Tween 20 (TBST). The PVDF membrane was soaked in the luminescent solution (ECL kit, WBULS0500, Millipore, Shanghai, China) and observed and photographed using an imager.

### 2.6. EdU Assay

C2C12 cells were seeded in a 96-well culture plate, with interference, overexpression, and corresponding control groups, each containing eight biological replicates. Once the cell density reached 80–90%, the EdU cell proliferation detection kit (RiboBio, Guangzhou, China) was used following the manufacturer’s instructions to prepare the click reaction solution and perform the corresponding experiments. Finally, the cells were observed and photographed using fluorescence microscopy.

### 2.7. Injection of Lentivirus-Encoded siRNA into Mouse Gastrocnemius Muscle

Lentiviruses encoding YTHDC1 interference RNA and control NC were synthesized by Suzhou GenePharma Co., Ltd. (Suzhou, China). The lentiviruses LV3-shNC and LV3-shYTHDC1 were diluted with 1× Dulbecco’s Phosphate-Buffered Saline (DPBS) to a concentration of 2 × 10^7^ TU/mL. Twelve six-week-old male C57 mice were used in the experiments. Each mouse received an injection of 100 μL LV3-shNC into the left gastrocnemius muscle of hind limbs and 100 μL LV3-shYTHDC1 into the right gastrocnemius muscle of hind limbs. The injections were administered weekly for one month, after which the mice were euthanized, and muscle samples were collected. Afterwards, the tissue was ground and RNA was extracted for real-time fluorescent quantitative PCR experiments and other experiments.

### 2.8. Transcriptome Sequencing and Identification of Alternative Splicing Events

C2C12 cells were seeded into six-well culture plates, and control (PC group) and experimental (OV group) groups were prepared with three biological replicates each. Overexpression and blank vectors were transfected according to the procedure described in Section 2.3. After three days of differentiation induction, total RNA was extracted for transcriptome sequencing. The sequencing was performed by Beijing Novogene Bioinformatics Technology Co., Ltd. (Beijing, China).The rMats statistical method was used to classify and analyze differential alternative splicing events in RNA-Seq samples. The expression level of alternative splicing events was defined by the inclusion levels. The difference in inclusion levels between the two groups was calculated to obtain the *p*-value, and the false discovery rate (FDR) was adjusted for multiple hypothesis testing. Events with FDR < 0.05 were considered differential alternative splicing events [33].

### 2.9. Hematoxylin–Eosin Staining

Paraffin sections were dewaxed, stained with hematoxylin and eosin, dehydrated, and sealed using a hematoxylin–eosin staining kit (APPLYGEN, Beijing, China). Finally, the samples were observed under a microscope, and the images were acquired and analyzed. ImageJ (1.8.0) was used to analyze the cross-sectional area of muscle fibers, take the average of sample images, and conduct comparative analysis.

### 2.10. Gel Electrophoresis

After overexpressing YTHDC1 in mouse C2C12 cells, total RNA was extracted and reverse-transcribed into cDNA (as described in Section 2.4). Alternative splicing was detected by PCR using 2× Taq PCR StarMix (GenStar, ZA012-101S) under the following conditions: 40 amplification cycles, followed by electrophoresis on a 3% agarose gel. All primer sequences are listed in Table S1. Gel electrophoresis images were captured by Tanon-3500 digital gel image system. The inclusion level of SE events was quantified using ImageJ software, and calculated as upper band intensity/(upper band intensity + lower band intensity).

### 2.11. Data Statistical Analysis

Each experimental group included three biological and three technical replicates. Real-time fluorescent quantitative PCR results were analyzed using the 2^–ΔΔCT^ method. Statistical analyses and significance tests were performed using Excel and GraphPad Prism (version 8.3.0), with a *t*-test or paired t-test applied for comparisons. The experimental data are presented as mean ± standard error of the mean. A *p* < 0.05 was considered statistically significant (*), and a *p* < 0.01 was considered highly significant (**).

## 3. Results and Analysis

### 3.1. YTHDC1 Inhibited Myoblast Proliferation

**Figure 1:**
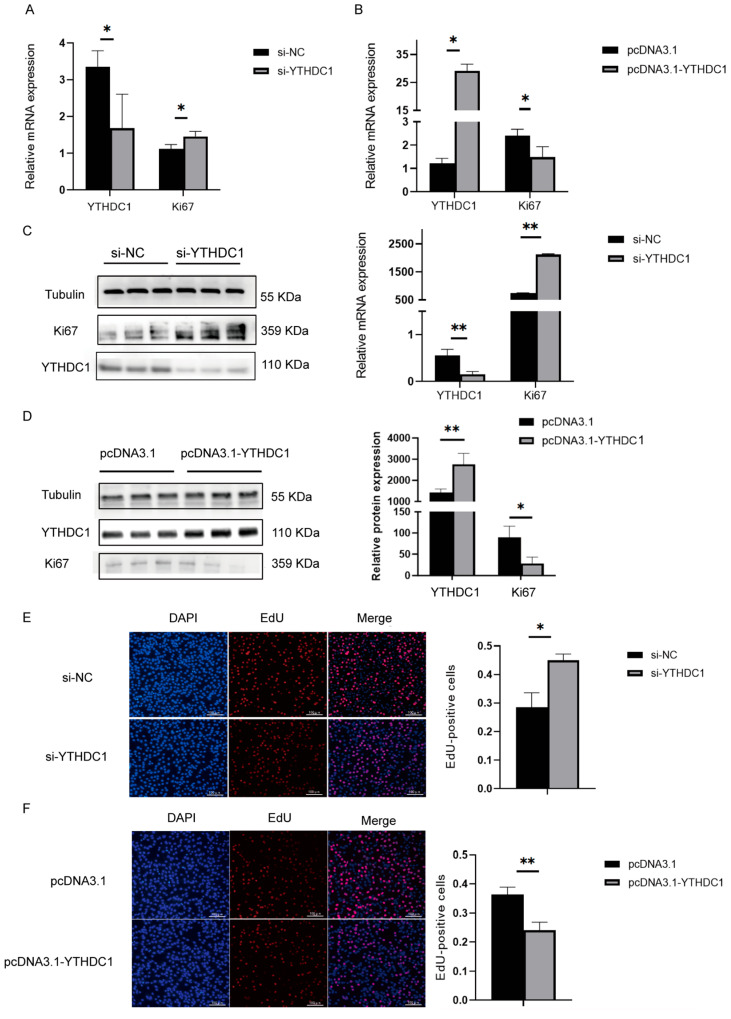
Effect of YTHDC1 on C2C12 cell proliferation as detected by real-time fluorescent quantitative PCR, Western blotting, and EdU assays. (**A**) Relative mRNA expression of interference YTHDC1. (**B**) Relative mRNA expression of overexpression YTHDC1. (**C**) Relative protein expression of interference YTHDC1. (**D**) Relative protein expression of overexpression YTHDC1. (**E**) EdU-positive cells of interference YTHDC1. (**F**) EdU-positive cells of overexpression YTHDC1. The scale is 100 μm; * *p* < 0.05; ** *p* < 0.01.

To assess the interference efficiency of YTHDC1 and its effect on the proliferation of C2C12 cells, we constructed interference siRNA and overexpression vectors. After transfecting the YTHDC1 interference fragment for three days, the mRNA expression level of YTHDC1 in C2C12 cells significantly decreased. In contrast, the mRNA expression levels of the muscle proliferation marker Ki67 were significantly increased (Figure 1A). After transfection with the overexpression vector, the mRNA expression of YTHDC1 was significantly increased in C2C12 cells, whereas the mRNA expression levels of the muscle proliferation marker Ki67 were significantly reduced (Figure 1B). Western blotting results indicated that after the interference of YTHDC1 expression, the protein expression level of Ki67 was significantly elevated (Figure 1C). Conversely, after YTHDC1 overexpression, the protein expression of Ki67 significantly decreased (Figure 1D). This result was confirmed by the EdU proliferation assay, as inhibiting YTHDC1 in C2C12 cells led to a significant increase in cell proliferation (Figure 1E) while overexpressing YTHDC1 significantly reduced cell proliferation (Figure 1F). These results suggested that the m^6^A recognition protein YTHDC1 inhibited the proliferation of C2C12 cells.

### 3.2. YTHDC1 Promoted Myoblast Differentiation

**Figure 2:**
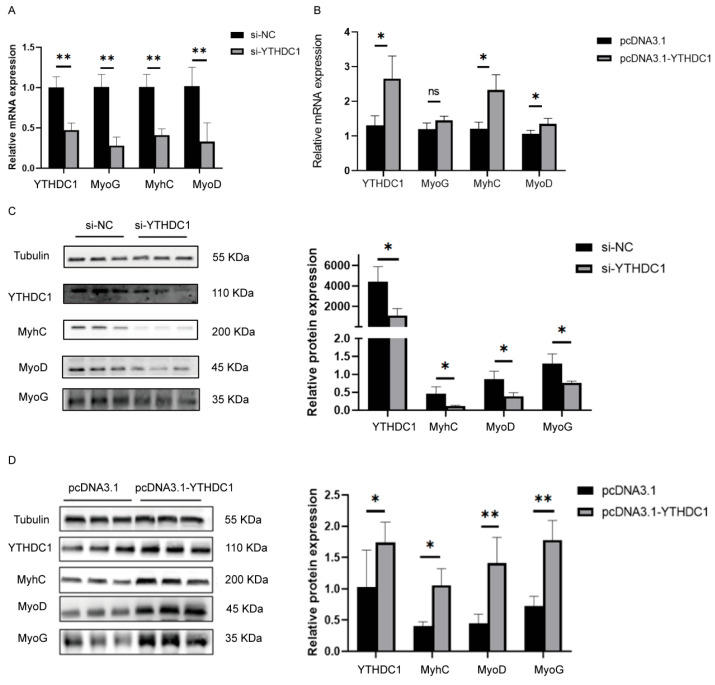
Effect of YTHDC1 on C2C12 cell differentiation assessed by real-time fluorescent quantitative PCR and Western blotting. (**A**) Relative protein expression of interference YTHDC1. (**B**) Relative mRNA expression of overexpression YTHDC1. (**C**) Relative protein expression of interference YTHDC1 (**D**) Relative protein expression of overexpression YTHDC1. ns, no significance, * *p* < 0.05; ** *p* < 0.01.

To investigate the effect of YTHDC1 in cell differentiation, we interfered with YTHDC1 expression in C2C12, and the mRNA transcription levels of differentiation marker genes MyoG, MyhC, and MyoD were significantly decreased (Figure 2A). Conversely, after transfecting with the overexpression vector for YTHDC1 and inducing differentiation, the mRNA expression levels of YTHDC1 and the differentiation marker genes MyhC and MyoG were significantly increased (Figure 2B). Western blotting results demonstrated that inhibiting YTHDC1 expression significantly promoted the protein expression of MyoG, MyhC, and MyoD in C2C12 cells (Figure 2C), whereas overexpressing YTHDC1 promoted the protein levels of MyhC, MyoD, and MyoG (Figure 2D). These results indicated that YTHDC1 promoted C2C12 cell differentiation.

### 3.3. Interfering with YTHDC1 Promotes Postnatal Muscle Growth in Mice

**Figure 3:**
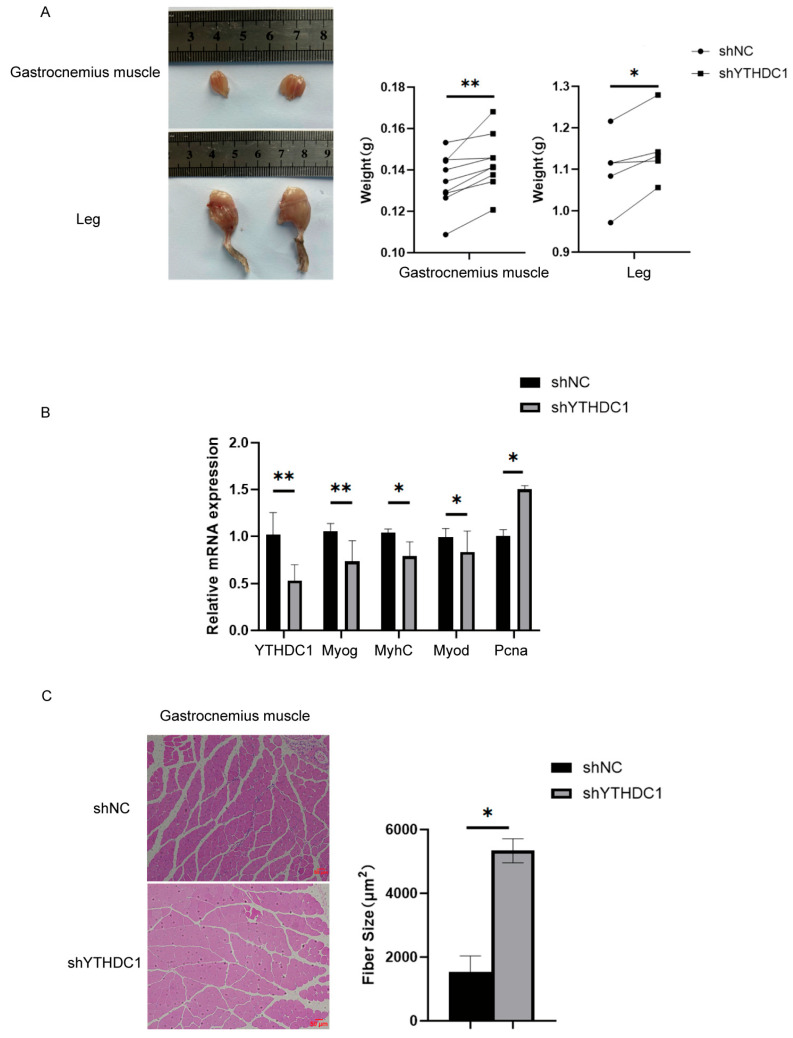
Detection of postnatal muscle growth in mice following interference with YTHDC1 using real-time fluorescent quantitative PCR and hematoxylin–eosin staining. (**A**) Changes in muscle volume and weight following interference with YTHDC1. (**B**) Relative mRNA expression of interference with YTHDC1. (**C**) Effects of YTHDC1 knockdown on muscle fiber cross-sectional area. Scale bar = 50 μm. * *p* < 0.05; ** *p* < 0.01.

To evaluate the effect of YTHDC1 interference on muscle weight in mice, we performed a mouse lentiviral injection experiment in twelve mice. The results demonstrated that after intramuscular injection of lentivirus to interfere with YTHDC1 expression, the volume and weight of the mice’s hind limbs significantly increased, with a marked increase in the gastrocnemius muscle volume and weight (Figure 3A). Figure 3B presents the results from RNA extracted from the gastrocnemius muscle of the left and right hind limbs of the mice one month after lentiviral injection. Real-time fluorescent quantitative PCR analysis of MyoD, MyoG, MyhC, and Pcna mRNA expression revealed that after significant interference with YTHDC1 expression in the mouse gastrocnemius muscle, the mRNA levels of differentiation markers were significantly decreased, while the proliferation marker Pcna was significantly increased. Moreover, histological examination using hematoxylin–eosin staining revealed a significantly increased cross-sectional area of muscle fibers in the gastrocnemius muscle of the interference group compared to that in the control group (Figure 3C). This indicated that interference with YTHDC1 promoted an increase in the cross-sectional area of muscle fibers in the gastrocnemius muscle of mice. These results were consistent with the in vitro cell culture findings, muscle weight changes, and real-time fluorescent quantitative PCR analysis of muscle tissues, further confirming that interference with YTHDC1 promoted the growth of skeletal muscle in mice.

### 3.4. Comparative Transcriptome Analysis of YTHDC1 Overexpression and Control Group Cells

**Figure 4:**
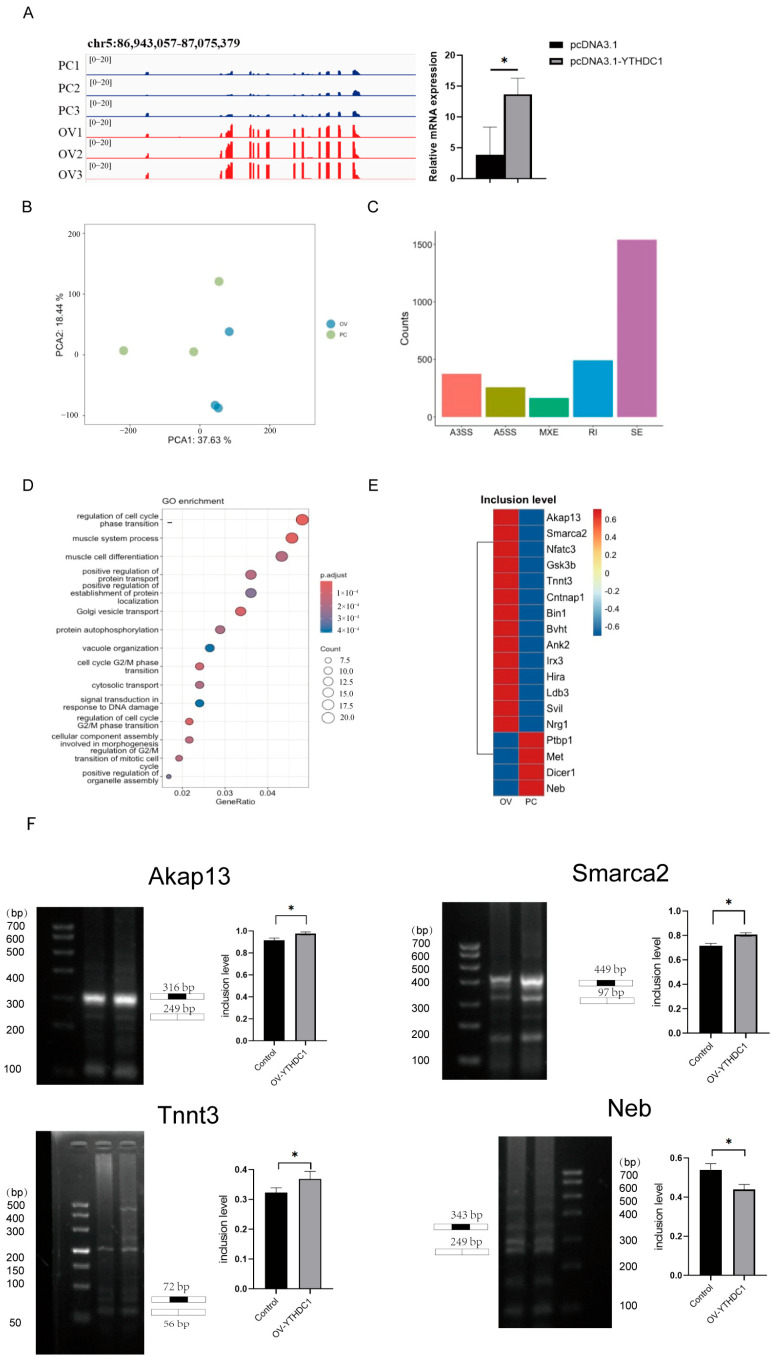
Comparative analysis of transcriptomes between YTHDC1 overexpression and control groups. (**A**) Efficiency of YTHDC1 overexpression in submitted samples. (**B**) PCA (Principal Component Analysis) of the PC and OV groups. (**C**) Classification and quantification of differential alternative splicing events. A3SS, alternative 3′ splice site; A5SS, alternative 5′ splice site; MXE, mutually exclusive exons; RI, retained introns; SE, exon skipping. (**D**) GO functional enrichment analysis. (**E**) Statistical analysis of differentially expressed genes. * *p* < 0.05. (**F**) The exon skipping events were validated by gel electrophoresis. * *p* < 0.05.

To further explore the regulatory factors at the transcriptome level during the development of mouse myogenic cells, we performed transcriptome sequencing analysis on the overexpression YTHDC1 experimental group and control group cells. Real-time fluorescent quantitative PCR results and sequencing data confirmed that YTHDC1 was significantly expressed in the samples sent for sequencing (Figure 4A). The principal component analysis plot of PC and OV groups demonstrated strong biological repeatability between the groups (Figure 4B), rendering it suitable for subsequent bioinformatics analysis. Using the rMats software (4.1.2) with a cutoff of FDR ≤ 0.05, we screened for differential alternative splicing events, focusing on their types and numbers. The findings indicated that exon skipping (SE) was the predominant form of alternative splicing, comprising approximately 50% of the total alternative splicing events, which was significantly higher than that of other types (Figure 4C). This suggested that SE was more prevalent in mouse myogenic cells.

After identifying the number and types of alternative splicing events in PC and OV groups, we performed a gene ontology (GO) functional enrichment analysis of the genes involved in the differential SE events (Figure 4D). The most significant GO terms were related to cell cycle regulation, muscle system processes, muscle cell differentiation, and positive regulation of protein transport. We further analyzed the muscle-related pathways associated with muscle system processes and muscle cell differentiation. The analysis revealed that 18 muscle development-related genes had altered splicing events in their transcripts (Figure 4E), including *Akap13*, *Smarca2*, *Tnnt3,* and *Neb*, which were validated by gel electrophoresis (Figure 4F), most of which play important roles in muscle development, such as *Tnnt3*, *Smarca2,* and *Neb* [34,35,36]. These findings suggested that YTHDC1 may regulate the alternative splicing of muscle development-related genes, thereby influencing skeletal muscle growth and development.

## 4. Discussion

This study used C2C12 cells as a model to investigate the regulatory role of YTHDC1 in skeletal muscle growth. C2C12 cells, as precursors of mouse skeletal muscle, have strong proliferation and differentiation potential. When induced with horse serum, they can rapidly differentiate into myotubes and express relevant marker genes [37,38,39]. Interfering with YTHDC1 expression in C2C12 cells increased the mRNA and protein levels of the proliferation markers Ki67 and Pcna, whereas YTHDC1 overexpression produced the opposite effect. Interfering with YTHDC1 expression in vivo increased the skeletal muscle weight and volume of the gastrocnemius muscle, and real-time fluorescent quantitative PCR results of muscle tissue aligned with the results of in vitro cell experiments. These findings suggested that the m^6^A reader protein YTHDC1 can inhibit myogenic cell proliferation and skeletal muscle growth. However, different m^6^A methylases played different roles in myogenic proliferation in earlier studies. Research has revealed that METTL3 can inhibit the expression of muscle-specific miRNAs, leading to muscle hypertrophy in mice [40]; METTL3 deletion results in muscle atrophy and impaired proliferation of C2C12 cells [41]. During the early stages of muscle injury, the METTL3/14 complex accelerates the proliferation of skeletal muscle stem cells by upregulating the expression of a mitogen-activated protein kinase interacting kinase [42]. However, recent experiments have demonstrated that METTL14 overexpression during early muscle injury inhibits myoblast proliferation [32]. These findings indicated that m^6^A modification bidirectionally regulates myogenic proliferation, with effects varying by methylation context and reader protein specificity [32,43,44].

Currently, the marker factors for skeletal muscle cell differentiation include MyoD (Myogenic Differentiation 1), MyoG (Myogen), and MyhC (Myosin Heavy Chain) [45]. MyoD is a myogenic determination factor that promotes the commitment of myogenic cells, while MyoG is a myogenic regulatory factor involved in myoblast fusion and differentiation [46]. MyhC is the main component of myosin and is a crucial part of myofibrils [32], which is highly expressed during the late stages of differentiation and is used to mark terminal myotube differentiation [44].Therefore, MyoD, MyoG, and MyhC are often used as marker genes for detecting cell differentiation. During early skeletal muscle differentiation, MyoD and MyoG are highly expressed and regulate myogenic differentiation [47]. Studies have found that downregulation of MyoD and MyoG leads to impaired or absent differentiation of myogenic cells in mice, which would cause abnormal muscle development and even death [48,49,50]. Research has indicated that fat mass and obesity-associated protein (FTO) functions as a demethylase and can reduce the m^6^A methylation modification of the growth inhibitory factor GADD45B mRNA and promote myogenic cell differentiation in sheep and chickens [51,52]. As m^6^A methylases, ALKBH5 and METTL14 show differential effects: interference of ALKBH5 significantly promotes bovine myoblast differentiation [32], while interference of METTL14 significantly inhibits bovine myoblast differentiation and decreases the mRNA and protein expression levels of differentiation markers, including MyhC, MyoD, and MyoG [53], whereas its homolog, METTL3, inhibits C2C12 cell differentiation [54]. Our research revealed that interfering with the YTHDC1 expression in C2C12 cells significantly reduced the mRNA and protein expression levels of myogenic differentiation markers, MyoD, MyoG, and MyHC. Conversely, YTHDC1 overexpression exhibited the opposite effect. In vivo, interference with YTHDC1 expression in mice also resulted in a significant decrease in the mRNA expression levels of MyoD, MyoG, and MyhC, consistent with the cell culture results. Collectively, these results establish that the m^6^A reader protein YTHDC1 promotes myogenic differentiation in both C2C12 cells and mouse skeletal muscle.

To investigate the effects of YTHDC1 overexpression on gene expression in C2C12 cells, we performed transcriptome sequencing analysis. The results demonstrated that SE represents the predominant alternative splicing event in murine myogenic cells, exerting significant biological influence. Notably, SE has been previously associated with Duchenne muscular dystrophy (DMD) pathogenesis [55]. The DMD gene on the X chromosome leads to abnormal dystrophin production, causing muscle atrophy in skeletal, cardiac, and smooth muscles [56]. Current therapeutic strategies primarily focus on inducing SE in the DMD gene to restore functional dystrophin [57], with SE of exons 44 or 45 shown to downregulate atrophy-related proteins and alleviate symptoms [55,58]. GO enrichment analysis of differentially expressed genes revealed that YTHDC1 overexpression significantly alters muscle differentiation-related gene expression, while the prevalence of SE events further suggests an underlying regulatory mechanism mediated by alternative splicing. The expression levels of muscle development-related genes enriched in these functional pathways also exhibited corresponding changes. In this study, after overexpression of YTHDC1, the levels of exon inclusion in transcripts of *Akap13*, *Smarca2*, *Tnnt3,* and other genes increased, while the levels of SE in transcripts of *Neb* and other genes increased. Studies have indicated that *Akap13* regulates immunological, reproductive, and skeletal muscle system activities, and *Akap13* knockout leads to thinning of the myocardium and severe cardiac developmental defects, causing death in mice [36]. *Smarca2* affects gene expression and cell function, and its alternative splicing generates complex functions for the BRAHMA (BRM) protein it encodes [36]. BRM knockout causes cultured cells to stop proliferating [59]. *Tnnt3*, a fast skeletal muscle troponin, regulates skeletal muscle growth and development [35]. Experimental evidence has confirmed that *Tnnt3* regulates muscle contraction and relaxation through ATPase activity in muscle filaments, and its overexpression promotes the differentiation of myogenic cells in goats and mice [34]. Conversely, splicing variations or copy number variations of *Neb* lead to early-onset distal myopathy [35]. Since the expression levels of genes related to muscle cell differentiation pathways were upregulated after YTHDC1 overexpression, it was preliminarily concluded that YTHDC1 may regulate the expression of muscle development-related genes through an alternative splicing process, which inhibited myogenic cell proliferation and promoted cell differentiation, thereby affecting skeletal muscle growth and development. However, the specific regulatory mechanisms of YTHDC1 in this process require further investigation.

In summary, YTHDC1 plays a role in regulating m^6^A modification in myoblasts, mediating its promotion of myoblast differentiation and inhibition of proliferation. At the same time, YTHDC1 also participates in regulating selective splicing events within the nucleus of myoblasts.

## 5. Conclusions

This study demonstrated that the m^6^A reader protein YTHDC1 inhibited myogenic cell proliferation, promoted cell differentiation, and facilitated skeletal muscle growth in mice. YTHDC1 also contributed to the increase in muscle fiber area. Transcriptome sequencing analysis preliminarily concluded that YTHDC1 may influence skeletal muscle growth and development by regulating the alternative splicing of muscle development-related genes, including *Akap13*, *Smarca2*, *Tnnt3*, and *Neb.*

## Figures and Tables

**Table 1:** Cell transfection system with Lipofectamine 3000.

Components	The Total System
Solution A.	400 μL Opti-MEM + 16 μL Lipofectamine 3000
Solution B.	400 μL Opti-MEM + 12.8 μL p3000 + 6 μg YTHDC1 Overexpression Plasmid/Empty Vector Plasmid

## Data Availability

The original contributions presented in this study are included in the article/Supplementary Materials. Further inquiries can be directed to the corresponding author(s).

## Supplementary Materials

The following supporting information can be downloaded at: https://www.mdpi.com/article/10.3390/ani15131978/s1. Figure S1: pcDNA3.1 (+) plasmid profile, Table S1: Primer sequence.
