# Supplementary material for: Effect of m6A Recognition Protein YTHDC1 on Skeletal Muscle Growth
Source: Animals (Basel). 2025 Jul 5;15(13):1978. doi: 10.3390/ani15131978 (PMC12248823; doi:10.3390/ani15131978)

Table S1：Primer sequence

Primers	Primer pairs
mus-MyoG	F:CCATCCAGTACATTGAGCGCCTACA<br>R:ACGATGGACGTAAGGGAGTGCAGAT
mus-MyoD	F:CGAGCACTACAGTGGCGACTCAGAT<br>R: GCTCCACTATGCTGGACAGGCAGT
mus-MyhC	F: CAAGTCATCGGTGTTTGTGG<br>R: TGTCGTACTTGGGCGGGTTC
mus-Ki67	F:ATCATTGACCGCTCCTTTAGGT<br>R:GCTCGCCTTGATGGTTCCT
mus-Pcna	F:TTTGAGGCACGCCTGATCC<br>R:GGAGACGTGAGACGAGTCCAT
mus-β-actin	F: GCCTCACTGTCCACCTTCCA<br>R: AGCCATGCCAATGTTGTCTCTT
mus-GAPDH	F: AGAACATCATCCCTGCATCC<br>R: GGTCCTCAGTGTAGCCCAAG
YTHDC1	F: GACTGGATTTGCAGGCGTGA<br>R: GGGGCATGGTGCTGGTAGTA
Akap13	F: GTTCAATGCGAGCTCTTG<br>R: GATGCTGTCTCTGGTCTG
Tnnt3	F: GAAGGAGGAAGTCCAAGA<br>R: CTTCCTCGTCCTCCTCCC
Smarca2	F: TAAATCTAGAGGCGGTTC<br>R: GTATCAATGATGGCGTTC
Neb	F: CCGAGCTAAGGTTTTCTT<br>R: ATCCCGACCACTGTCACT
si-YTHDC1	F: GUCGGUUACAGAAUAUAAATT<br>R: UUUAUAUUCUGUAACCGACTT
siRNA-NC	F: UUCUCCGAACGUGUCACGUTT<br>R: ACGUGACACGUUCGGAGAATT

Figure S1：pcDNA3.1 (+) plasmid profile

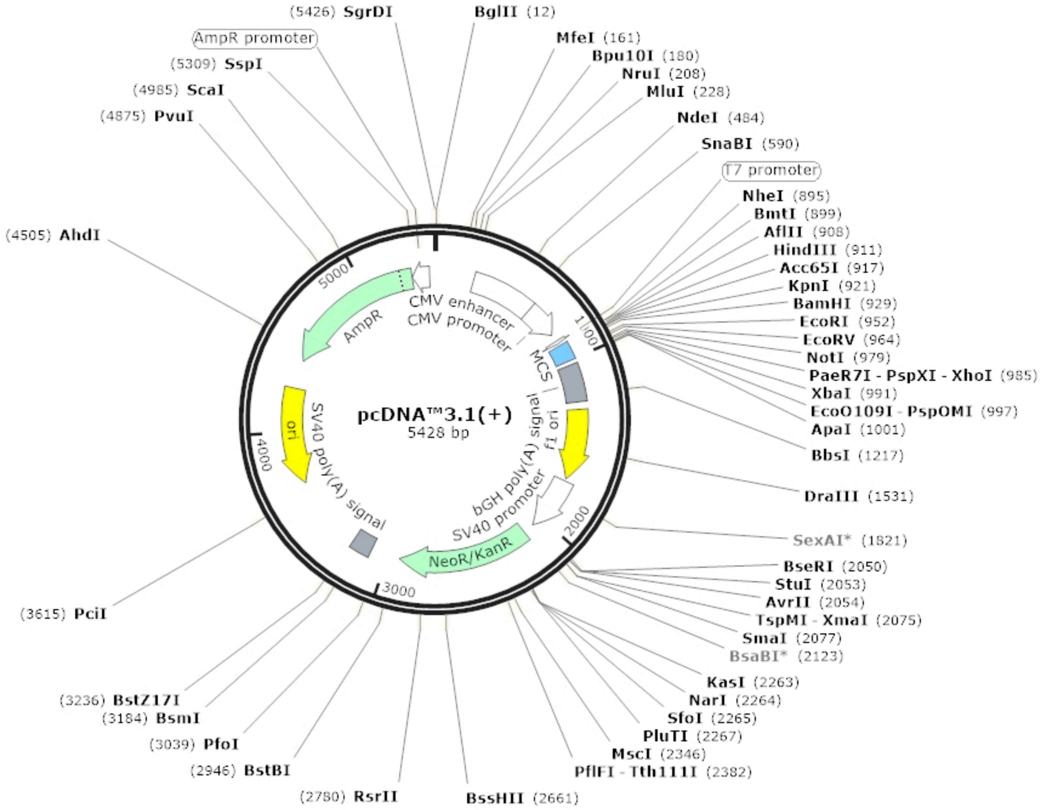

Supplement: Supplementary file 1 [file animals-15-01978-s001.zip › animals-3680607-supplementary.pdf]
